# Web Content Accessibility of Consumer Health Information Web Sites for People with Disabilities: A Cross Sectional Evaluation

**DOI:** 10.2196/jmir.6.2.e19

**Published:** 2004-06-21

**Authors:** Xiaoming Zeng, Bambang Parmanto

**Affiliations:** ^1^Department of Health Information ManagementSchool of Health and Rehabilitation SciencesUniversity of PittsburghPittsburgh PAUSA

**Keywords:** People With Disabilities, World Wide Web, Internet, Health Services Accessibility

## Abstract

**Background:**

The World Wide Web (WWW) has become an increasingly essential resource for health information consumers. The ability to obtain accurate medical information online quickly, conveniently and privately provides health consumers with the opportunity to make informed decisions and participate actively in their personal care. Little is known, however, about whether the content of this online health information is equally accessible to people with disabilities who must rely on special devices or technologies to process online information due to their visual, hearing, mobility, or cognitive limitations.

**Objective:**

To construct a framework for an automated Web accessibility evaluation; to evaluate the state of accessibility of consumer health information Web sites; and to investigate the possible relationships between accessibility and other features of the Web sites, including function, popularity and importance.

**Methods:**

We carried out a cross-sectional study of the state of accessibility of health information Web sites to people with disabilities. We selected 108 consumer health information Web sites from the directory service of a Web search engine. A measurement framework was constructed to automatically measure the level of Web Accessibility Barriers (WAB) of Web sites following Web accessibility specifications. We investigated whether there was a difference between WAB scores across various functional categories of the Web sites, and also evaluated the correlation between the WAB and Alexa traffic rank and Google Page Rank of the Web sites.

**Results:**

We found that none of the Web sites we looked at are completely accessible to people with disabilities, i.e., there were no sites that had no violation of Web accessibility rules. However, governmental and educational health information Web sites do exhibit better Web accessibility than the other categories of Web sites (P < 0.001). We also found that the correlation between the WAB score and the popularity of a Web site is statistically significant (r = 0.28, P < 0.05), although there is no correlation between the WAB score and the importance of the Web sites (r = 0.15, P = 0.111).

**Conclusions:**

Evaluation of health information Web sites shows that no Web site scrupulously abides by Web accessibility specifications, even for entities mandated under relevant laws and regulations. Government and education Web sites show better performance than Web sites among other categories. Accessibility of a Web site may have a positive impact on its popularity in general. However, the Web accessibility of a Web site may not have a significant relationship with its importance on the Web.

## Introduction

The World Wide Web (WWW) has become an increasingly essential resource for health information consumers. One recent study estimated that 73 million US residents searched for health information online during the year 2002 [1]. The investigators estimated that seventy-odd percent of the online population search for health-related information for their decision-making [1]. Eysenbach and Kohler [2] estimated that approximately 4.5% of all search queries submitted to Web search engines are health related, which is equivalent to a global minimum of 6.75 million health-related searches on the Web every day. With the advances of computer and Internet technology, the distribution of the online population is becoming representative of the general population in terms of demographic and socioeconomic status [3].

The ability to obtain accurate medical information online quickly, conveniently, and privately provides health consumers with the opportunity to make informed decisions and participate actively in their personal care [4]. Little is known, however, about whether this online information is equally accessible to people with disabilities who must rely on special devices or technologies to process online information due to their visual, hearing, mobility, or cognitive limitations.

The latest report on Internet use from the National Telecommunication and Information Administration (NTIA) demonstrated that people of all ages, races, and ethnicities, including people with disabilities, are moving more and more of their activities online [3]. A recent investigation on Internet use by people with disabilities reported that people without disabilities are four times more likely (38.1%) to use the Internet than are people with disabilities (9.9%) [5]. Similar patterns remain even when factors, such as income, gender and educational attainment, are taken into account [5]. The large disparity in Internet usage may be attributable to problems with the accessibility of Web content [5]. Nielsen (2001) reported that the usability of the Web is about three times better for users without disabilities than it is for users with disabilities [6].

For people with disabilities, the Web is very often the only source of information that they may access without having to depend unduly on others. Equivalent Internet access to health information will open a door to people with disabilities by offering them exciting possibilities for independent living and community participation [7]. People with disabilities can find a wealth of information on the Internet that addresses many issues of special concern to them, including chronic disease information and rehabilitation and assistive technology services [8]. According to a recent report, people with disabilities tend to seek health related information online more frequently than the able-bodied population [9]. Nevertheless, for health information Web sites to be of real use to people with disabilities, they must first be accessible to them. Health information Web sites are a classic example of the "inverse information law": access to appropriate information is particularly difficult for those who need it most [4].

### Background and Prior Work

Web content accessibility helps people with disabilities access Web pages directly or use assistive technologies. Many people with disabilities have to rely on specialized software or hardware to access the Web. For example, people who are visually impaired have to install a software package called a screen reader to read all the content on the Web page aloud to them. Some people who are blind also use a talking browser like IBM Home Page Reader to access the Web page aurally. Some people who are blind prefer a hardware-level solution like the computer-controlled Braille embosser to help them perceive content of the Web page haptically. Regardless of the solution favored by the users with disabilities, if the content of the Web page is not available to their remaining sensory channel, then the page is not accessible to them.

The Web inadvertently has become increasingly inaccessible to people with disabilities as it adopts numerous emerging multimedia technologies. The Web at its beginning was designed for sharing and accessing documents across different computer systems and platforms. These documents are primarily text-based and mostly accessible to assistive technology, such as screen readers. With the introduction of appealing multimedia content, however, the Web is becoming an information medium that is not accessible to or not easily interpreted by assistive technology. Graphics, animations, and even video/audio clips, now commonly appear on the Web. The absence of alternative information about multimedia content makes them less accessible to people with disabilities than those with multimodal access to the multimedia content. The rapid expansion of e-commerce also makes the Web even more complicated and less accessible for people with disabilities. As Herbert A. Simon [10] once stated, "What information consumes is rather obvious: it consumes the attention of its recipients. Hence a wealth of information creates a poverty of attention, and a need to allocate that attention efficiently among the overabundance of information sources that might consume it." Web page developers believe that multimedia content could lure more visitors to the Web site and make them stay longer. However, they may overlook or ignore the accessibility for people with disabilities to that multimedia content because its primary purpose is to draw attention from potential consumers, the majority of whom are not people with disabilities.

Realizing this dilemma, the World Wide Web Consortium (W3C), the international organization that oversees the standardization and operation of the Web, announced the establishment of the Web Accessibility Initiative (WAI) on April 7, 1997 [11]. Supported by all W3C members, including such heavyweight stakeholders as Microsoft and IBM, the WAI plays a central role in promoting and correcting the functionality of the Web for people with disabilities. The first major responsibility of the WAI was to formalize guidelines for Web content developers and designers. WAI introduced Web Content Accessibility Guidelines (WCAG) to the public as a draft in 1998, and developed it into a full recommendation in 1999 [12]. WAI expanded the guidelines to be applicable in the design of user agents (e.g., Web browsers or assistive technology agents like the screen reader JAWS for Windows), authoring tools (e.g., Microsoft FrontPage or Macromedia Dreamweaver) and related techniques, and a practical checklist [13,14].

There are two basic themes reflected in the WCAG: ensuring graceful transformation of Web pages, and making content understandable and navigable. By providing Web pages that transform gracefully, people with disabilities or users with device limitations will be able to access them without constraints. Keys to graceful transformation include separating structure from presentation, providing text equivalents to non-textual elements, creating documents that work even if the user cannot see and/or hear, and creating device-neutral documents. When the content is understandable and navigable, end users can utilize the page in a more effective, efficient and satisfactory manner. Keys for making content understandable and navigable include providing a navigating context and orienting information, providing a clear navigation mechanism, and ensuring succinct content descriptions.

Another initiative in the development of accessibility standards is Section 508, conducted by the US Access Board [15]. The Access Board issued standards for accessible information technology under the Reauthorized Rehabilitation Act. These amendments strengthen Section 508 of the Rehabilitation Act of 1973. It mandates that when federal agencies develop, procure, maintain, or use electronic and information technology, they shall ensure that the electronic and information technology will allow federal employees with disabilities access to and use of the same information and data as that accessed and used by federal employees who are not individuals with disabilities, unless an undue burden would be imposed on the agency. Section 508 also mandates that agencies ensure equal access to individuals with disabilities who are members of the public seeking information on data that are comparable to that provided to those who are not individuals with disabilities, unless undue burden would be imposed on the agency. Section 508 clearly defines the accessibility for people with disabilities for federal government Web sites. Section 508 took effect on February 20, 2001.

Many software packages have been developed and commercialized to help Web developers evaluate the accessibility of their Web sites to people with disabilities [16]. These packages can scan Web pages, list computer detectable violations of Web accessibility standards, and give warnings for suspicious HTML snippets. Some tools integrate themselves into Web site developing or quality control programs to assist Web developers in quickly eliminating the inaccessible parts. Bobby, one of the earliest and most well known packages for checking Web accessibility, was used in our study.

Researchers from different disciplines have evaluated Web accessibility and usability of Web sites in various domains. The Journal *Library Hi Tech* published two special issues dedicated to Web content accessibility of Web-based information resources for people with disabilities [17,18]. Axel Schmetzke [19] maintains a Web accessibility survey site that aspires to be a clearinghouse for studies involving the collection of accessibility data pertaining to Web sites and online resources in education. The site listed many Web accessibility evaluation studies on libraries and higher education Web sites. Another related effort is the Web Usability Index (WUI), a free Web usability statistics database provided by UsableNet [20]. It employs an automatic Web usability evaluation tool for testing Web accessibility and obtains daily statistics of Web usability of sample Web sites from the Internet. According to WUI, only about 43% of current Web sites provide excellent or good Web usability design.

Although the Web is considered a powerful force for reshaping the healthcare infrastructure, the accessibility of Web content to people with disabilities is not a primary consideration for most designers of Web sites providing health related information [21]. Very few research studies have been conducted on the accessibility of health information Web sites for people with disabilities. Research studies on the accessibility of health information Web sites are, for the most part, about the find-ability and search-ability of Internet Web sites by online search engines or about the availability of information technology for the people who need it 22-26]. Previous guidelines related to the quality of health information Web sites failed to emphasize the accessibility of Web sites by people with disabilities [27] until the National Cancer Institute (NCI) published research-based guidelines addressing Web usability [28]. Chapter 3 of the NCI report is specifically dedicated to the issue of Web accessibility for persons with disabilities although the rest of the guidelines can also benefit general Web users.

The only study known to us that covers health information Web sites was the study conducted by Joel Davis in 2002 [29]. Davis explored the extent to which Internet-based health information is accessible to visually impaired individuals who rely on automated screen readers. Davis selected 500 individual Web sites representing 50 common illnesses and conditions for evaluation. The study found that accessibility is currently very low-only 19% of the examined sites' home pages were accessible. It also found that the reason for the inaccessibility of the Web pages was noncompliance with the recommended design and coding changes.

Our study will be different from other studies in several ways: first, the study will check the degree of accessibility not only of home pages (main pages) of health information Web sites, but also of other Web pages within certain levels below the home pages. Second, the majority of other studies report the state of accessibility in terms of the absolute number of violations of accessibility checkpoints. Although absolute numbers of violations of Web content accessibility provide useful information about the state of accessibility, it is not straightforward for direct comparison of general accessibility between Web sites, and it does not include the complexity of the webpage into the evaluation. Third, we will investigate the relationship between Web accessibility and other features of a Web site including function, popularity and importance.

### Research Questions

The overall objective of the study was to evaluate the accessibility of consumer health information Web sites for people with disabilities. We were interested in the following specific research questions:

1. What is the current level of accessibility for consumer health information Web sites?
  We were interested in using automated computer programs to evaluate the current state of content accessibility of Web sites providing health information to consumers. The checkpoints used in the program were derived from Web accessibility specifications -- WCAG 1.0 and Section 508.
2. What is the relationship between Web accessibility and the functional category of the Web site?
  We were interested in determining the distribution of the level of accessibility among these Web sites after we categorized them into functional groups. We expected government and education Web sites to provide information that is more accessible to consumers than other types of Web sites because of the existing specifications and initiatives.
3. What is the relationship between Web accessibility and the popularity of the Web site?
  The hypothesis for this research question is that there is a positive correlation between the degree of Web accessibility and the popularity of the Web sites. The variable representing popularity of a Web site was determined by its visiting traffic.
4. What is the relationship between Web accessibility and the importance of the Web site?

We wanted to investigate whether there is any correlation between the level of Web accessibility and the importance of the Web site. We expected to find that more important Web sites would be more accessible to people with disabilities. The variable representing the importance of a Web site was determined by the page importance ranking data provided by a Web search engine.

## Materials and Methods

### Design

The study is a cross-sectional study concentrating on the degree of accessibility of Web sites providing consumer health information. We used established Web accessibility specifications as the sources for constructing the measurement framework. Additionally, we investigated the relationship between Web accessibility and other features including function, popularity, and importance.

### Materials

An individual Web site providing consumer health information is the unit of analysis in the study. Because the exact number and distribution of Web sites are not pre-determinable due to the tremendous size and rapid growth of the Web, probability based sampling methods, such as random or stratified sampling, are not applicable in the study. An alternative sampling approach widely adopted by researchers conducting studies on Web sites is to use search engines or online Web site directories.

We acquired a list of consumer health information Web sites from the directory service of the Google search engine (See Appendix A). Google's directory service obtained data from the Open Directory Project, the largest, most comprehensive human-edited directory of the Web [30]. We included all Web sites under the subdirectory "Health/Resources/Consumer" as our candidate Web sites for evaluation. These are health information Web sites for the public, and their content are not necessarily specific to issues related to disability. We excluded ones that had their content changed to non-health related areas or were continuously unavailable during our study period after we reviewed the home page of each Web site.

After selecting the sample Web sites, we needed to establish a limit to the scope of the Web pages to be included within each site. Because WCAG only applies to Web pages, other content formats such as PDF (Portable Digital Format) files were not considered. However, server side scripting such as Active Server Page (ASP), or JavaServer Page (JSP) is able to dynamically produce HTML-based code at the client side, therefore we took these types of pages into consideration. Second, we needed to determine the number of Web pages from each Web site to be included in the analysis. Due to the large number of Web pages in some Web sites, it was not feasible to include all the pages into the study. We selected only the first two layers from the home page within a domain of a Web site in our sample. We hypothesized that the first two layers would be the most visited and would reflect the overall accessibility of the Web site for the study. The other reason for choosing only the first two layers was that Bobby version 4.01 has the ability to only process a limited number of pages on a given Web site because it consumes a large amount of computer memory during the analysis. When we selected three layers from the home page, Bobby encountered an "out of memory" error when analyzing large Web sites using a Pentium 2.4Ghz desktop computer with 1Gb memory.

### Measurements

#### Web Content Accessibility

One of the objectives of the study is to construct a measurement framework to assess the accessibility of consumer health information Web sites. As we discussed in the background section, two major specifications served as the normative guidelines for Web content accessibility design. The first-the W3C Web Content Accessibility Guideline 1.0 (WCAG)-is a stable international specification developed through a voluntary industry consensus. The US Access Board published the second specification-Electronic and Information Technology Accessibility Standards-in December 2000, pursuant to the US rulemaking process as required by Section 508 of the Rehabilitation Act Amendments of 1998 [31]. Both specifications offer checklists or rules that Web developers should follow with regard to content accessibility for people with disabilities. These two specifications largely overlap; only three of the checkpoints defined in Section 508 are not mentioned in the WCAG guideline 1.0. WCAG is more comprehensive than Section 508 on checkpoints of Web content accessibility, and it provides a priority level to each checkpoint to reflect severity of violations. Therefore, WCAG was used as the foundation for the accessibility metrics we developed.

The number of violations of each checkpoint is a component of our scoring method called the Web Accessibility Barrier (WAB) score. For example, a Web page with fewer accessibility checkpoint violations, e.g., providing an alternative description for an image object, would be considered to present fewer barriers for people with disabilities and will have a lower WAB score.

Because we are interested in automated evaluation of the degree of accessibility of a Web site, the subset of Web accessibility checkpoints demanding manual checking are not included in the calculation of the WAB score. For example, compliance to the rule "If you use color to convey information, make sure the information is also represented another way," cannot be verified until a manual check is done. For a list of Web accessibility rules that need to be manually checked, please see the WAI references [32].

WCAG attaches a three-point priority level to each checkpoint from its impact on Web accessibility. Priority 1 checkpoints mandate the largest level of compliance while Priority 3 checkpoints are optional for Web content developers. In weighting the calculation of the WAB score, we used the priority level in reverse order. The weighting factor for Priority 1 violations is 3, for Priority 2 violations is 2, and for Priority 3 violations is 1.

Using only the number of violations of Web accessibility checkpoints, however, may bias the results of the measurement. For example, a Web page with five "image without alternative text" violations may have 500 image objects embedded in the page and the Web page with one "image without alternative text" violation may have only one image object in the page. The developer of the first page may have already paid a great deal of attention to and put great effort into complying with the Web accessibility specifications while the developer of the second page may be completely unaware of accessibility. Therefore, the number of true violations of a checkpoint must be normalized against the number of potential violations of the checkpoint. In the last example, true violations are the image objects without alternative text, and the potential violations include all image objects on the page. Whenever a Web developer puts an image element into a Web page, he increases the potential that there could be a violation of the "alternative text" checkpoint. Table 1 explains the selection of potential violations from HTML code. The average WAB score of all Web pages within a Web site will be the WAB score of the Web site.

**Figure 1:**
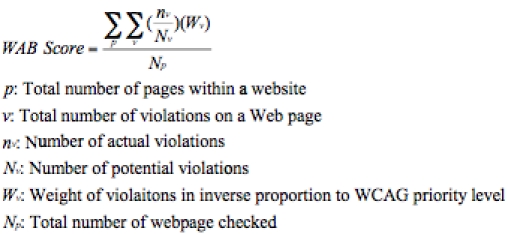
Formula for Calculating the Web Accessibility Barrier (WAB) Score

Figure 1 summarizes the calculation of the WAB score of a Web site as a formula. A higher score means there are more accessibility barriers on the site, while a lower score indicates fewer barriers. A score of zero denotes that the Web site does not violate any Web accessibility guidelines and should have no automatically detectable accessibility barriers to people with disabilities.

We employed several program tools to examine the true and potential violations of the Web pages. Bobby is a checking program that can examine a Web page and report violations of Web accessibility checkpoints [33]. It is the most widely used accessibility checking software package and has been around longest. Bobby was originally developed by the Center for Applied Special Technology [34], and is now maintained and distributed by Watchfire Corporation [35].

Bobby desktop version 4.0.1 was used in this study. The desktop version can check compliance with WCAG of an entire Web site or only certain layers from the main page of the Web site. The version 4.0.1 can check non-compliance issues with both WAI and Section 508 checkpoints. After checking a Web site, Bobby generates a report in eXtensible Markup Language (XML) format that can be further processed to extract data about true violations.

Bobby implements 91 distinct testing rules, each of which maps onto a specific WCAG checkpoint. The Bobby tests are classified into a number of different "checking" categories, as follows: (1) Full: Bobby automatically checks this rule and decides whether there is an error. (2) Partial: Bobby automatically performs some checking of the rule, but cannot decide the existence of violations. Instead, the line number is used as a warning to the testers. (3) Partial Once: Similar to the Partial category, but the warning is not specific to an individual line. (4) Ask Once: Bobby does not have a mechanism to check the rule, so the rule is presented as a reminder to the testers.

For all categories other than Full, a human tester must manually evaluate the Web site further to determine the WCAG compliance, which is not viable for a large scale Web site study like this one. We used only the 25 rules that Bobby implements with Full checking capacity for our evaluation. Even for the rules with "Full" checking capacity, we still could not determine the quality of the compliance with WCAG. For example, the Web page developer could simply put the file name of the image into the "alt" attribute of the <IMG> element to avoid a flag from Bobby. The quality of such compliance is much less acceptable than providing detailed description in the "ALT" attribute.

The data of corresponding potential violations for each checkpoint can be extracted using a Web crawler program, which is an automated program that follows hyperlinks to visit Web pages. We developed a lightweight Java-based Web crawler program to access Web pages at remote Web sites and determine the number of potential violations of Web accessibility checkpoints. We did not use the built-in Web crawler in Bobby because it cannot be customized to check potential violations of checkpoints in a Web page. We also made use of the "homemade" crawler as the basis for future development of tools for Web accessibility evaluation. For a list of rules for extracting data of potential violations, please see Table 1. Since the crawler embedded in Bobby and the "homemade" Web crawler may retrieve an unmatched number of pages for the different capacities of both crawlers, we only used the Web pages retrieved by both programs in the study.

**Table 1:** Checkpoints and the Determinant of the Number of Potential Violations

WAI Priority	Checkpoint	Determining the number of potential violations
1	Provide alternative text for all images.	All <img> elements
1	Provide alternative text for each APPLET.	All <applet> elements
1	Provide alternative content for each OBJECT.	All <object> elements
1	Provide alternative text for all image-type buttons in forms.	All <input type="image" …> elements
1	Provide alternative text for all image map hot-spots (AREAs).	All <area> elements
1	Each FRAME must reference an HTML file.	All <frame> elements
1	Give each frame a title.	All <frame> element
2	Use a public text identifier in a DOCTYPE statement.	1*
2	Use relative sizing and positioning (% values) rather than absolute (pixels).	All <table>, <th>, <td>, and <frame> elements
2	Nest headings properly.	All heading elements
2	Provide a NOFRAMES section when using FRAMEs.	All <frameset> element
2	Avoid blinking text created with the BLINK element.	Same as the number of true violations#
2	Avoid scrolling text created with the MARQUEE element.	Same as the number of true violations#
2	Do not cause a page to refresh automatically.	1*
2	Do not cause a page to redirect to a new URL.	1*
2	Make sure event handlers do not require use of a mouse.	Number of event handler for both keyboard and mouse
2	Explicitly associate form controls and their labels with the LABEL element.	Number of form elements such as <input>, <select>, and <textarea>
2	Create link phrases that make sense when read out of context.	Number of <a> elements
2	Do not use the same link phrase more than once when the links point to different URLs.	Number of <a> elements
2	Include a document TITLE.	1*
3	Client-side image map contains a link not presented elsewhere on the page.	Number of <area> elements
3	Identify the language of the text.	1*
3	Provide a summary for tables.	Number of <table> elements
3	Include default, place-holding characters in edit boxes and text areas.	Number of <input type = "text">, <text area>, and <select> elements
3	Separate adjacent links with more than white space.	Number of links

^*^ This feature is determined at the entire page level. Therefore, we assign 1 to the number of potential violations.

^#^ The number of potential violations of this feature was not able to be determined. Therefore, we used the same number of the true violations as the number of potential violations. The frequency of the violations is simply 0 or 1 according to the formula of Web Accessibility Barrier (WAB) score.

##### Function of the Web Sites

We measured three variables-function, popularity and importance-as other features of the Web sites. We classified the candidate Web sites based on their functions. We used a taxonomy that classifies the Web sites into six functional categories: e-commerce, corporate, portal, community, government, and education. We derived the taxonomy from a similar one from the Web Usability Index database [20]. An e-commerce Web site conducts online transactions of health related products or services. A Corporate Web site represents a health care service corporation online. A Portal Web site provides entrance to various health related information resources. A Community Web site hosts online activities for patients or health information seekers. Government and education Web sites have the postfix ".gov" and ".edu", respectively in their domain names. Table 2 lists example Web sites from each category.

**Table 2:** Example Web Sites of Each Functional Category

Category	Definition	Examples
Portal	Web site provides entrance to various health related information resources	Web MD (http://www.webmd.com)
Government	Web site has the postfix ".gov" in the domain name	Health Finder from U.S. Department of Health and Human Services (http://www.healthfinder.gov)
Corporate	Web site represents a health care service corporation online	Mayo Clinic (http://www.mayoclinic.com)
E-commerce	Web site conducts online transaction of health related products or services.	Health Windows (http://www.healthwindows.com)
Community	Web site hosts online activities for patients or health information seekers.	Health Forum (http://www.healthforum.com)
Education	Web site that has the postfix ".edu" in the domain name	HealthLink from medical college of Wisconsin (http://healthlink.mcw.edu)

Two evaluators individually assigned each Web site to one of the aforementioned categories. In case of a disagreement about the assignment, both evaluators discussed it until reaching a consensus. Each Web site fell into only one of the categories. Government (.gov) and education (.edu) Web sites had precedence over other function categories. For example, HealthFinder.gov is a government Web site, but its function is also to provide health information as a portal. We assigned it to the government instead of portal category. The reason for the precedence is that we were especially interested in the degree of Web accessibility of these two functional categories because of the existing specifications and initiatives.

##### Popularity of the Web Sites

We used daily traffic-ranking data of each Web site that was provided by the search engine Alexa as the measurement variable for the popularity of the Web sites [36]. Alexa calculates statistics about the traffic patterns of a Web site after aggregating visit data from all users who install Alexa's toolbar in their Web browsers during a three-month period. Because the Alexa toolbar is currently only available for Microsoft Windows and Internet Explorer, the accuracy of the traffic ranking of the Web site is limited. However, it may reflect the popularity of the Web site on the Web to a certain extent. We retrieved the ranking data of the entire candidate Web sites from Alexa on February 25, 2003.

##### Importance of the Web Sites

We measured the degree of importance using the PageRank score of each Web site available from the Google search engine. The PageRank score relies on the uniquely hypertext nature of the Web by using its vast link structure as an indicator of an individual page's value. In essence, Google interprets a link from page A to page B as a vote by page A for page B. Therefore, the PageRank score of a page can be viewed as an indicator of the importance of the page. But Google looks at more than the absolute volume of votes, or links that a page receives; it also analyzes the page that makes the vote. Votes cast by pages that are themselves "important" weigh more heavily and help to make other pages "important." [37] Because Google does not provide PageRank in a numerical value from its searching interface, we had to rank the sites according to an implicit PageRank score and use the ranking number as the value of the variable of importance. We retrieved the ranking of importance of all candidate Web sites from Google on February 26, 2003.

#### Data Analysis

All statistical analyses were performed with alpha value at 0.05 and power at 0.80. Descriptive statistics (means and standard deviation) were calculated for each variable considered in the study. Univariate statistics of the WAB scores were calculated at the level of each category. Then a one-way ANalysis Of VAriance (ANOVA) test was applied to the WAB scores at the level of the Web site's functional category. If the ANOVA test indicated a large difference in the WAB scores among different categories, the post hoc Bonferroni test of the WAB scores between different categories was conducted. The alpha level was adjusted for multiple comparisons in the Bonferroni test.

Google ranked Web sites with a sub-category from highest to lowest PageRank value. Therefore, we used the ranking sequence as the value of Web page importance for nonparametric Spearman correlation. Nonparametric Spearman correlation statistics were also conducted to measure the level of correlation between the WAB scores and the popularity of the Web sites. All statistical analyses were conducted using the SPSS 11.0 software package.

### Results

#### Descriptive Statistics

The Google subdirectory "Health/Consumer/Resources" lists 122 Web sites, 14 of which were excluded because their content are no longer healthcare related or they were not active during the study period. The assessing program retrieved 7,109 Web pages from the remaining 108 sites. Means and standard deviations of WAB scores for the remaining 108 Web sites were calculated. The average WAB score was 9.31 with standard deviation of 6.29. None of the 108 Web sites was absolutely accessible (WAB score = 0). The National Institutes of Health (NIH) Combined Health Information Database (CHID) Web site (http://chid.nih.gov/) achieved the lowest WAB score, i.e., it had the fewest accessibility barriers, of the sites tested (0.97), while a community Web site (http://www.discussyourhealth.com/) received the highest WAB score (24.99). The five most frequently violated WCAG checkpoints of all webpages were: "identify language of the text" (77.0%), "use a public text identifier in a DOCTYPE statement" (65.6%), "provide a summary for tables" (61.6%), "use relative sizing and positioning (% values) rather than absolute (pixels)" (60.0%), and "provide alternative text for all images" (52.2%).

#### WAB and Categories

Among the six functional categories of Web sites, government Web sites were most accessible and had the lowest WAB scores, and portal Web sites were least accessible to people with disabilities, indicated by higher WAB scores (Table 3).

**Table 3:** Means and Standard Deviations of the Web Accessibility Barrier (WAB) Scores Across Functional Categories

Category	Mean	Number of Web sites (n)	Standard Deviation
Portal	13.17	30	6.16
Government	1.42	6	0.39
Corporate	9.03	25	3.94
E-commerce	8.53	8	3.39
Community	9.92	29	6.8
Education	2.06	10	1.16
Total	9.31	108	6.29

**Figure 2:**
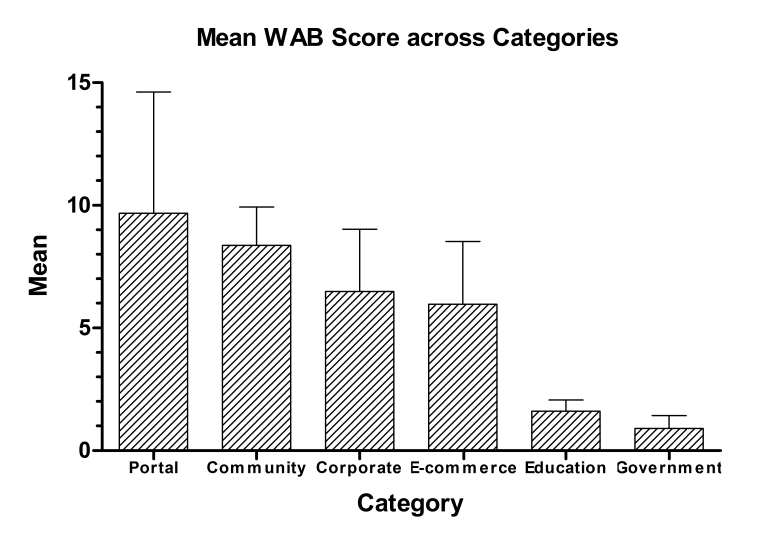
Means of the Web Accessibility Barrier (WAB) Score of Each Category. Height of Each Bar Represents Mean WAB Score. The Horizontal Tick Above Each Bar Represents Standard Deviation of WAB Score

The average scores of Web accessibility were calculated for each of the Web categories and the results indicate possible clustering among the six categories, as shown in Figure 2.

Statistically significant differences among the category groups were found using the ANOVA test on the WAB scores (F = 9.705, P < 0.001). In addition, the post hoc Bonferroni test found that the mean WAB scores of governmental and educational Web sites were significantly different from the rest of the categories (P < 0.001). There is no statistically significant difference between any two categories within each of the two clusters.

#### WAB Score vs. Popularity and Importance

Furthermore, the Spearman correlation test indicates a statistically significant, though modest, correlation between the WAB score and the Alexa traffic ranking (*r*= 0.28, P < 0.01). No statistically significant correlation between the WAB score and the PageRank of Web sites was found (*r*= 0.15, P = 0.111) using the Spearman correlation test (Table 4). The correlation between the Alexa's traffic ranking and Google's PageRank was statistically significant (*r*= 0.32, P < 0.01) using the Spearman correlation test.

**Table 4:** Spearman Correlation Coefficients Between the Web Accessibility Barrier (WAB) Score, Alexa Ranking and Google's Pagerank

	WAB score	Alexa ranking	PageRank
WAB score	1.00	0.28*	0.15
Alexa ranking	0.28*	1.00	0.32*
PageRank	0.15	0.32*	1.00

^*^ Correlation is significant at the 0.01 level (2-tailed). The complete results data set is included as a data supplement with this article.

### Discussion

Awareness of accessibility issues is increasing among developers of Web sites due to law enforcement, public initiative, and prospective commercial incentives [21]. Even though many evaluation tools are now available to developers intending to improve the accessibility of their Web sites, the status of Web accessibility, especially among health information Web sites, is largely unknown. Compliance with the specifications of Web content accessibility is necessary to narrow the digital divide between the information affluent and digitally underserved people, in this case, those with disabilities. Ours is the first study to address the issue. It provides a relatively comprehensive evaluation of the Web accessibility of consumer health information Web sites, and proposes a metric evaluation for measuring the accessibility of a Web site, taking into account both accessibility violations and the complexity of the Web site presented as potential violations of accessibility checkpoints. This approach provides a more accurate and impartial measurement about the level of accessibility barriers than using only the absolute number of violations as has been employed by most other evaluations. Additionally, the study investigates the relationship between the level of accessibility and the function, importance and popularity of a Web site.

#### Current Level of Web Accessibility Across Consumer Health Information Web Sites

No consumer health information Web sites satisfied all of the Web accessibility requirements, which may be attributed to Web site developers knowing little about accessibility standards, the lack of effective and efficient evaluation and repair tools, and the pressure to update information on the Web site quickly. Web accessibility, if ever considered, is often an afterthought once Web content design is finished. This implies that program tools that produce efficient, effective post-hoc repairs of Web content accessibility violations, or an accessible proxy server that transforms and filters inaccessible online content for people with disabilities may be more accepted by both the developers and Web site visitors.

#### Web Accessibility and Functions of the Web Sites

Of the sites providing health information, government sites followed by education sites are the most accessible. This compliance may be attributed to Section 508, since it is mandatory for all federal agencies [38]. High compliance among sites that fall under this mandate also indicates that legal activities would facilitate the removal of accessibility barriers for people with disabilities.

None of the tested Web sites, including the most accessible government sites, passed the WCAG guideline priority 1 checkpoints, even though the five most frequently violated checkpoints have technically uncomplicated solutions if designers pay attention to them. This may imply that the Web site editor simply overlooked the errors and, for such editors, an automatic Web site monitoring program could be very helpful in identifying and correcting these errors. Other possible reasons for such imperfection are the lack of integrated accessibility tools or functions within Web site editing software. Most Web site editing tools make it optional to strictly follow accessibility rules.

The education Web sites are the second most accessible category. Section 508 is not strictly mandatory for the information technology available on educational Web sites, but high awareness of WCAG rules and legal requirements on most campuses may contribute to better accessibility among the education Web sites. Furthermore, although Section 508 does not mandate all education Web sites, it does apply to educational programs and projects that receive federal funding, as many do, which may explain the high compliance to WCAG rules among education sites.

#### Web Accessibility and Popularity of the Web Sites

The accessibility of a Web site also correlates with its popularity, possibly implying that people with disabilities are more likely to visit sites that contain fewer or no barriers to them. A more accessible Web site may be more usable for the general population because it can also improve the efficiency, effectiveness, and ease of using the Web site [39]. Meanwhile, accessible Web pages will have better opportunities for indexing by Web search engines, which use programs called crawlers to access Web pages on the Internet and store Web page indexes in a database for fast Web information retrieval. Web crawlers work similarly to Web users who are blind and using screen reader programs. Therefore accessible Web pages will have more chances to be indexed by a Web crawler [40]. Subsequently the overall popularity of the Web sites increase since they attract a group of visitors who have difficulties accessing other sites containing more Web accessibility barriers. Other reasons for the correlation between accessibility and popularity include the possibility that people may take notice that a Web site is accessible and tend to visit it often, or Web developers of accessible Web sites spend more time ensuring their Web sites are appropriate in following other usability rules that make visiting easier for the public.

#### Web Accessibility and Importance of the Web Sites

The correlation between Web accessibility and a Web site's importance was not statistically significant in our study, although the correlation between its importance and popularity was statistically significant. The measurement of the importance of a Web site was derived from comprehensive link analysis on the Web. It revealed the value of the Web site by measuring how many and what kind of other Web sites link into it. It does not necessarily reflect the value of other HTML elements, especially those Web accessibility related elements. A Web site can be very important in terms of PageRank because many other Web sites have links to it, even though it is not accessible to persons with disabilities when they directly visit it.

#### Limitations

Please note that there are several limitations to this study. First, although this study attempts to comprehensively assess the accessibility of a Web site, it is not practical for some Web sites, especially those with large numbers of archived documents. The Bobby program often freezes when checking all layers of a Web site, and this resulted in the decision to check only a manageable two layers of Web pages in this study. A more robust tool needs to be adopted or developed for future studies.

Second, only the checkpoints of Web accessibility that can be examined automatically by a computer program were studied. Many other checkpoints require a manual check of pages to ensure the compliance of the content with the guidelines of Web accessibility. WAI proposed a comprehensive framework for evaluating Web content accessibility which requires multiple steps involving several evaluation tools to ensure the accuracy of the evaluation results. Although this type of evaluation is important for quality assurance of individual Web sites, the cost of such a large operation makes it impractical for an evaluation study involving many Web sites. This study assumes that the checkpoints that can be automatically evaluated will strongly correlate to the manual checkpoints and can be used as a surrogate assessment for accessibility of a Web site. Future studies might explore the agreement between these two groups of checkpoints.

Furthermore, the traffic ranking information provided from Alexa may skew towards users of Internet Explorer on a Windows operating system, underestimating the traffic to sites that are disproportionately accessed by people using other browsers or operating systems. The site most likely to suffer from this bias is AOL (America Online), since their members commonly use AOL browsers to access the site.

The WAB score in the study can be used to measure the degree of accessibility of a site. However, it should not be used as the only indicator for Web accessibility, which includes other checkpoints that can not be automatically assessed by computer programs. An experienced Web developer can fine-tune a Web site to produce a perfect WAB score. However, this does not necessarily mean that the Web site is entirely accessible to people with disabilities when they visit it.

### Conclusions

This study evaluates the current state-of-accessibility of consumer health information Web sites for people with disabilities. Accessibility barriers are present in all site categories, especially commercial Web sites. Government and education Web sites show better performance than those in other categories. Accessibility may have an impact on its popularity because people with disabilities will feel more comfortable visiting those sites with fewer accessibility barriers. This study attempts to increase the awareness of Web accessibility among the designers of consumer health information Web sites.

## Appendix A

### Consumer Health Web Sites Selected from Google for Accessibility Evaluation

**Table A1:** Consumer Health Web Sites Selected from Google for Accessibility Evaluation

Name	Address	Description	Category	Alexa Rank	Web Accessibility Score	Page Rank
Consumer Reports Online	http://www.consumerreports.org	Information and advice on health products, services, and decisions.	community	2,015	3.44	4
Health A to Z	http://www.healthatoz.com/	Includes a directory of more than 50,000 professionally-reviewed Internet resources, supportive online communities, and a calendar.	community	22,436	2.33	8
Dr. Gabe Mirkin	http://www.drmirkin.com	Reports on health, fitness, and nutrition news from talk show host Gabe Mirkin, M.D., in text and audio form.	community	52,851	3.56	28
Body1.com	http://www.Body1.com/	Health news and medical information community for consumers.	community	211,705	6.99	30
ProWho	http://www.prowho.com/	Locate health professionals anywhere in the world.	community	1,098,188	6.91	34
MDAdvice.com	http://www.mdadvice.com/	Provides health and medical information, health tips, resources, experts, news, chats, and community support.	community	88,689	11.09	45
Askapatient.com	http://www.askapatient.com/	Provides a database of patient opinions and ratings of medicine effectiveness. Also includes weekly consumer opinion polls on healthcare topics, and a health care research assistance section.	community	424,906	3.22	46
Health & Family Resource Guide	http://www.noeasytask.com	Personal and professional sites containing valuable information and links.	community	871,197	2	48
1UpHealth: Your Health Resource on the Net	http://www.1uphealth.com/	Offers information concerning condition and diseases. Listed by alphabet, systems/types, and by demography.	community	57,395	5.67	51
CountryNurse.com	http://www.countrynurse.com	Includes information on clinics, family wellness, disease prevention, diet, exercise and pharmacies.	community	2,742,182	3.45	58
Selfhealth	http://www.selfhealth.com.au/	Consumer health information including a medical Q&A database and an Australian drug database. Covers infertility, emotional health, sexual health & integrative & complementary health issues.	community	1,055,493	4.87	59
Oneday MD Program	http://www.onedayMD.com	A medical e-course, written for the everyday layman. Easy to understand and digest.	community	ND	7.98	60
Wonderful World of Diseases	http://www.diseaseworld.com/	Catalog of links and information on diseases and human conditions. Includes an online bookstore.	community	375,309	4.99	63
Medidoctor	http://www.medidoctor.com/	A home health guide to diagnosis and treatment, and when to see your doctor or go to hospital.	community	603,475	11.98	65
Wellness.com	http://www.wellness.com	Includes health resources, discussion and news.	community	403,791	13.99	72
Digital City - Health	http://www.digitalcity.com/health/	Health resources and providers across the United States.	community	1,030	24.88	77
Discuss Your Health	http://www.discussyourhealth.com/	Discussion forums and health information.	community	ND	24.99	81
Mindy Machanic's Change Pages: Wellness and Health Info	http://www.mindymac.com/Health.html	Articles on healthy foods, cancer and breast cancer. Includes comprehensive links to additional resources for health and wellness.	community	3,156,154	5.99	83
Health-Center.com	http://www.health-center.com/default.htm	Resources on numerous health topics. Includes a bulletin board and discussion forum.	community	49,607	5.44	86
C.S.S. Doctor's Credentials Search	http://www.tese.com/css/index.html	Search for a Doctor's Medical School, Board Certification, residence training, licensing, disciplinary action (if any), and other important information.	community	668,459	10.34	88
Health In Depth	http://www.healthindepth.com/	Health information links to newspapers, magazines and internet resources.	community	ND	14.98	89
American Care	http://www.americancare.net/	Provides access to a network of medical professionals and medical facilities.	community	ND	20.99	93
DoctorInfo	http://www.maxpages.com/doctorinfo/	Provides searches for background information on medical doctors or doctors of osteopathic medicine.	community	3,232	21.66	98
Internet Health Library	http://www.health-library.com	Searchable index to healthcare sites.	community	247,229	15.89	103
Urgent Medical Help	http://www.urgentmedicalhelp.com/	Offers a list of medical topics, and expert advice.	community	ND	16.88	108
Health+Plus / Dr. David Clark [1]	http://communities.msn.com/DrDavidClarkHealthPlus	Information and alternatives on various health challenges.	community		4.66	109
Well-aware	http://www.well-aware.co.uk	Provides information on conditions, complementary treatments and expert views, all written by doctors in the United Kingdom.	community	279,876	6.77	110
AOL Anywhere Health Web Channel: Tests and Tools	http://search.aol.com/dirsearch.adp?query=health%20tools	Informational question and answer tool for assessing your personal health and fitness. Addresses a variety of common conditions, diseases, and disorders.	community	23	10.44	112
SciTalk.com	http://www.scitalk.com/	Science related resources for the public on health and disease. Discussion boards, chat, news, patents, clinical trials and books.	community	831,828	11.21	113
			community			
Health Communication Network	http://www.hcn.net.au/	Provides the up-to-date health information on a variety of subjects.	corporate	62,522	5.928365085	21
BluePrint for Health	http://blueprint.bluecrossmn.com/	A health and wellness portal which provides health information, personalized newsletters and interactive health tools.	corporate	55,309	6.117271677	22
Apples For Health	http://www.applesforhealth.com/	Consumer news on healthcare topics.	corporate	109,078	2.770206902	26
The Health Resource, Inc.	http://www.thehealthresource.com/	Specialized medical research reports on mainstream, experimental, and alternative treatments, specialists, and support organizations.	corporate	751,741	6.473851872	35
HealthLink Plus	http://www.healthlinkplus.org/	Consumer health information on general health, health care providers, medical research, insurance, wellness, mental health, and alternative medicine.	corporate	1,012,019	9.223706688	49
HealthFrontier.com	http://www.HealthFrontier.com/	Offers information including diseases and conditions, nutrition, exercise, mental health, live discussions and a message board.	corporate	1,118,483	4.706849182	52
Accenthealth	http://www.accenthealth.com/	Database of news, articles, and information about conditions, medications, and tips for living a healthy lifestyle.	corporate	215,095	3.094589415	53
HealthStatus	http://www.healthstatus.com	Free reports on body fat percentage, body mass index, calorie burning activities, target heart rate and smoking costs. Online health risk assessment which provides resources based on your health risks.	corporate	100,176	14.48851992	55
Health-MD	http://www.health-md.net	Provides informational links covering all aspects of health.	corporate	1,894,244	14.28787455	57
eCureMe.com	http://www.ecureme.com/	Identify symptoms to make a self-diagnosis; set up online consultations with physicians and therapists; view online medical dictionary of diseases, treatments, drug information.	corporate	28,626	8.174559539	61
Patient Protect	http://www.patientprotect.com/en/	Medical consultation devoted to protecting and defend patients. Contributes to reducing health costs, by preventing abuses, negligences, medical errors and incompetence in the health field.	corporate	ND	14.26290622	62
A Second Opinion Medical Information Services	http://www.physicians-background.com	Medical treatment options, physician background check service, best hospitals and doctors. (Ft. Walton Beach, FL)[Fee based service - ed]	corporate	1,164,345	8.513670197	64
50+Health	http://www.50plushealth.co.uk	Health topics, lifestyle magazine, discussion forum, news and research.	corporate	986,902	8.035373745	66
Medical Elite	http://www.medical-elite.com/	International medical consulting and information company that specializes in locating medical specialists. Translated into English, Arabic, Chinese, Portuguese, Russian, Spanish, and other languages.	corporate	1,574,968	5.083424917	71
GetWell.org	http://GetWell.org/	Offers resources for consumers on medical conditions, treatment and research.	corporate	3,373,473	3.704081763	74
Global Health News and Resources	http://www.globalhealthnews.org/	Offers news and resources in the health industry.	corporate	ND	9.244440892	75
The Lifestyle Doctor	http://www.lifestyledoctor.uk.com/	Information on lifestyle issues and simple ways to help oneself.	corporate	3,097,649	12.39462059	78
UHealthy Network	http://www.uhealthy.com/	Global health information network and community that integrate every aspect of Health and Fitness in one place.	corporate	94,364	4.341446672	80
Vital Star Health, Science and Technology Resource Center	http://www.vitalstar.com/health.html	Free online Medical Check up How healthly your are? Test your eye, BMI, carbs, protein, cholestrol, heart, height, calories, depression. Plus articles, news and updates related to health and fitness.	corporate	1,154,166	11.10723414	84
WoundHeal.com	http://www.woundheal.com/info/infoIndex.htm	Educational information and resources for the non-surgical healing of pressure ulcers, at home.	corporate	2,266,566	14.12597302	85
Wellness Hour Medical Informational Talk Show	http://www.wellnesshour.com	A medical talk show aired in over 100 cities throughout the United States.	corporate	1,523,336	7.012615012	97
Health and Wellness	http://www.health-and-wellness.org/	Explains the importance of "wellness", information on how to rate your own wellness, and how onsite programs can boost employee productivity.	corporate	ND	13.80270648	104
Medical Information Dictionary	http://www.medical-information-dictionary-and-videos.com/	Dictionary with extensive listings on treatments. Current information on new medical procedures and definitions.	corporate	777,994	9.957871849	105
Discovery Health	http://www.discoveryhealth.com/	Offers news and a variety of health information resources.	corporate	495	7.237195863	121
			corporate		9.433343772	
HealthAnswers	http://www.healthanswers.com/	Contains health news and information, including a health encyclopedia.	corporate,portal	80,996	12.46205114	119
HealthWindows	http://www.healthwindows.com	A membership healthcare network that helps individuals to become more knowledgeable and active participants in managing their personal health.	e commerce	723,999	2.13913917	23
Quackbusters	http://www.quackbusters.com.au/	Offers nutrition and general health information.	e commerce	507,145	5.362357494	33
Clinnix: Health Care Information	http://www.clinnix.net	Includes daily news, travel information and disease management.	e commerce	802,713	13.12822279	40
Prozac Prescription Online Pharmacy	http://www.prozac-prescription-online-pharmacy.com	Consult this Prozac guide to get prices, medical facts and tips on where to buy. Includes interaction data and uses.	e commerce	2,776,050	15.78729775	68
HealthCheck Risk Assessment	http://www.bodybalance.com/hra/	Useful health risk assessment.	e commerce	606,674	15.26809823	69
Health Depot	http://blakkat.com/health.htm	Directory to health and medical sites about diet, fitness, disabilities, diseases, health resources, products and sales.	e commerce	148,973	15.04753096	76
Alternative Healing and Lifestyles	http://alternatehealing.com	Offering resources ranging from weightlifting to mind, body, and Nutrition. Many links to health sites.	e commerce	ND	15.50132577	100
DoctorOnNet.com [2]	http://www.doctoronnet.com/	Resources for information regarding medicine, doctors, health, fitness and related topics.	e commerce	445,255	8.931488165	102
			e commerce			
CNN Health	http://cnn.com/HEALTH/	Health news, chats and advice from CNN.	education	26	2	1
MCW HealthLink	http://healthlink.mcw.edu/	Features health news and information, produced by the Medical College of Wisconsin.	education	15,109	2.01	5
McGill Molson Medical Informatics: Student Projects	http://sprojects.mmi.mcgill.ca/	A growing collection of multimedia projects in medical teaching. Developed by McGill medical students under the supervision of the McGill Medical Faculty. Includes a student/faculty forum.	education	5,536	1.66	27
Evaluation of English and Spanish Health Information on the Internet	http://www.rand.org/publications/documents/interneteval/	The findings of a large study that describes and evaluates English and Spanish health information on the Internet. Assesses search engine performance and the quality and readability of health information on the Internet, and provides conclusions and recommendations.	education	25,966	4.56	31
Duke University Healthy Devil On-Line	http://gilligan.mc.duke.edu/h-devil/	Online medical resources and information.	education	4,808	3.44	32
Health Leader	http://www.uthouston.edu/hLeader/index.html	A webzine produced by The University of Texas Health Science Center, which provides information to help you make better decisions about your health.	education	563,631	2.44	36
BBC Online	http://www.bbc.co.uk/health/conditions/	Information about a wide range of health conditions, summaries of illnesses and treatments, and details of organizations that can provide medical and emotional help and support.	education	39	1.03	120
Your Health IS Your Business	http://weber.edu/hp/Faculty/molpin/bushea/index.html	Site includes information on health and wellness including primarily links to sites on the internet on health and wellness.	education	28,291	1.46	122
BBC Health Your Rights	http://www.bbc.co.uk/health/consumer/index.shtml	Advice on everything from finding the shortest waiting lists to what to do if you think you are a victim of medical negligence.	education	39	1	123
BBC News - Medical notes	http://news.bbc.co.uk/hi/english/health/medical_notes/	Information briefs on health topics related to the news, including several on environmental health topics. Listed alphabetically.	education	39	1	125
			education			
National Institutes of Health -- Health Information Index	http://health.nih.gov	Main consumer health information page for the National Institutes of Health (NIH)	government	286	1.45	2
Healthfinder (tm)	http://www.healthfinder.gov	Resource for consumer health and human services.	government	22,660	1.02	3
Combined Health Information Database	http://chid.nih.gov/	A database produced by health-related agencies of the Federal Government. Provides titles, abstracts, and availability information for health information and health education resources.	government	286	0.97	10
Agency for Health Care Policy and Research	http://www.ahrq.gov/consumer/	Consumer health and patient information on health plans and insurance, prescriptions, conditions and diseases, surgery, quality of care, quitting smoking, and prevention and wellness.	government	68,255	1.63	12
Michigan Electronic Library - Health Information Resources	http://mel.lib.mi.us/health/health-index.html	Extensive resources and links of interest to the health consumer and to professionals.	government	21,404	2.01	29
Buying Medical Products Online	http://www.fda.gov/oc/buyonline/	Information for consumers from the US Food and Drug Administration. How to determine if a site is legitimate; how to spot health fraud; and how to report fraudulent sites.	government	3,067	1.44	114
			government			
MayoClinic.com	http://www.mayoclinic.com/	Clinical experts provide current medical information and news on health topics.	portal	4,156	16.61	6
Dr. Koop's Community	http://www.drkoop.com/	Former Surgeon General Koop's resources for health information. A wide variety of topics, an encyclopedia, pharmacopeia, and resources guide.	portal	14,244	15.06	7
WebMD - Consumer	http://my.webmd.com/	Frequently updated portal for healthcare, chat forums, health quizzes, news and consumer product updates.	portal	514	14.10	9
Diseases, Disorders and Related Topics	http://www.mic.ki.se/Diseases/index.html	Karolinska Institutet, Stockholm, Sweden. Comprehensive listings of links to medical information, most reliable, some not.	portal	9,844	4.94	11
Achoo Healthcare Online	http://www.achoo.com/	Portal and directory for medical news and information.	portal	101,658	12.59	13
Medinex	http://www.medinex.com	Provides a safe health search for medical information available on the internet.	portal	504,726	14.96	14
Healthgrades.com	http://www.healthgrades.com/	Grades the performance of hospitals, physicians, health plans, nursing homes and other health care providers in the United States.	portal	71,775	22.90	15
Halls MD	http://www.halls.md/	Clinical calculators of body surface area, breast cancer risk and body mass.	portal	94,880	4.79	16
Health In Focus	http://www.healthinfocus.co.uk/	Independent UK health information and medical information resource.	portal	293,598	16.48	17
Laurus Health Information	http://www.LaurusHealth.com	Information on health conditions, pharmaceuticals, medical news, plus profiles of physicians and hospitals. Free registration.	portal	47,108	4.20	18
Cochrane Consumer Network	http://www.cochraneconsumer.com/	This international group dedicated to the study of evidence-based medicine, explains how to decipher clinical studies and how to use them when making decisions about medical care.	portal	688,474	21.34	19
Internet Pharmacy and Online Pharmacies Verification	http://www.nabp.net/vipps/intro.asp	National Association of Boards of Pharmacy provides searchable listings of approved online pharmacies.	portal	97,563	17.54	20
Medicine OnLine	http://www.meds.com	In-depth information on cancer for health care professionals and patients.	portal	87,469	12.95	25
Find a Doctor in Your Area	http://www.vab.com	Geographic directory of doctors with links to their web sites.	portal	352,920	20.31	37
Best Doctors	http://www.bestdoctors.com/	Comprehensive knowledge-based medical referral service.	portal	102,232	9.47	39
Health Consumer Alliance	http://www.healthconsumer.org	Provides information to consumers and advocates about access to health care for low-income consumers, including consumer education materials in 13 languages.	portal	2,026,586	7.49	42
Doctor Healthynet	http://www.doctorhealthynet.com/	Offers diagnosis and treatment of conditions and diseases, medical procedures, preventive health guidelines, and sources of free medicines.	portal	630,052	18.59	44
AnswerMed.com	http://www.answermed.com/	Provides basic information on medical conditions and procedures including symptoms, diagnosis, treatment, predicted outcome and alternative diagnoses.	portal	1,178,690	3.16	47
Health, Nutrition and Fitness	http://www.health-nutrition-and-fitness.com	Search this extensive directory of sites, focusing on exercise and fitness, nutrition, mental health, depression and therapy, and diseases such as osteoporosis.	portal	ND	7.37	54
Health Plug	http://www.healthplug.com/	Provides information on prescription drugs and other medications, with a message board and news links.	portal	2,136,121	8.01	67
MDinteractive	http://mdinteractive.com/	Providing consumers with healthcare information and resources in every medical specialty. Providing physicians and patients with an efficient way to create and store medical records interactively.	portal	2,494,795	9.17	79
Access Place Health	http://www.accessplace.com/health.htm	Link collection about medical news, health and fitness and some medical specialties.	portal	109,338	3.35	90
SymptomTracker	http://www.symptomtracker.com	An interactive medical diagnosis and treatment reference that uses brief yes/no questions about a users symptoms to arrive at possible conditions and treatments. [Please note the "Warning" before proceeding - ed]	portal	467,922	4.33	92
Surgery Door Home Healthcare Guide	http://www.surgerydoor.co.uk/HomeHealthcareGuide/	Symptoms of common illnesses and ailments. From the UK's on-line health service.	portal		18.73	95
iMedNetworks	http://www.imednetworks.com/	An internet-based healthcare network that connects physicians and patients to each other and to a virtual world of medical information, tools, and services.	portal	2,217,720	6.54	111
Germ Detectives [3]	http://www.germdetectives.com/	Portal site to health information, hoax busters, and ways of avoiding Bad Science.	portal		15.78	115
KnockDoctor.Com	http://www.knockdoctor.com	Health portal covering subjects such as family health, beauty, yoga, ayurveda, health and fitness.	portal	3,067,983	22.95	116
MedicalClub [4]	http://www.medicalclub.com	Provides interactive free health information on Womens, Childrens and Family health concerns. The site also includes extensive information on herbal medicines, supplements and First Aid. Bilingual, English/Spanish.	portal		17.81	117
Planetamber	http://www.planetamber.com/	Global International health, medical and disability resources database. Categorized medical condition search for people with disabilities or health impairments, their families and those providing services and support.	portal	2,783,436	10.16	124
Search-It-All	http://www.search-it-all.com/biomedical.asp	Doctor and hospital search, nutrition facts, drug and disease lookup and health information.	portal	4,129,642	5.40	126
			portal			
Mylifepath	http://www.mylifepath.com	Provides information on health and wellness, daily health news and message boards.	Site not available	410,709		24
Consumer Laboratory Testing Information	http://www.ascls.org/labtesting/index.htm	A thorough guide to medical laboratory tests, why they are performed, and what they might mean.	Site not available	933,912		41
Carepanion [5]	http://www.carepanion.com/	Provides life care products, services and tools. Contains links, news, articles and suggested further resources on medical issues.	Site not available	1,097,498		43
The Medical Information Warehouse	http://www.medfindnow.com/	Offers medical and disease information including poison control and child abuse areas.	Site not available	1,498,881		50
SearchPointe [6]	http://www.searchpointe.com/	Free verification of name, location, and education of doctors and chiropractors.	Site not available	3,423,196		56
HealthExpos.com	http://www.healthexpos.com/	Information on upcoming events and expos in Minnesota.	Site not available	ND		70
Health Forums	http://www.healthforums.com/	Customized libraries of health and well-being information. Log in to access an extensive library of resources.	Site not available	ND		73
LivingandHealth.com	http://www.livingandhealth.com/	Offers information on topics such as diabetes, irritable bowel syndrom (IBS), hypertension, and epilepsy.	Site not available	16,128		82
No Frills Health	http://www.nofrillsguide.com/health.htm	An easy to use and useful guide to health sites on the net.	Site not available	ND		91
Citypractice.com [7]	http://www.citypractice.com/	Provides information on preventitive approaches in physical, behavioural and emotional healthcare.	Site not available	ND		94
Health4m	http://www.health4m.com	Health forum for support, information, or exchanging ideas. Topics of discussion include general health, fitness, nutrition, diets, women's/teen's/men's issues, depression, A.A/N.A recovery, acne, mental illness.	Site not available	1,454,527		96
Worldnethealth.com	http://worldnethealth.com/	Offers an online medical encyclopedia with a large medical slide library and videos.	Site not available	ND		99
Health and Wellness Topic Center [8]	http://health.medscape.com/wellnesscenter	Provides information and interactive health management tools across a variety of disciplines.	Site not available	514		101
Journey To Wellness [9]	http://www.ihealthradio.com	African American health magazine and radio show. Listen to archived radio programs of the nationally syndicated radio programs, as well as read program related articles and link to credible related resources..	Site not available			106
Medicalresourcesusa.com	http://www.medicalresourcesusa.com/	Offers guides to American hospitals, health clinics, medical practices and specialties.		ND		38
Healing Action [10]	http://www.healingaction.com/	Health related articles on food, aging, ecology as it relates to health.		3,802,737		87
A Patient's Guide to the Internet	http://www.patientsguide.com	A step-by-step guide for patients seeking medical information on the Internet.		203		107
Building Better Health	http://www.pcsrx-online.com	Provides information on health and wellness, along with daily health news, full-text journal and magazine articles.		4,103,022		118

## Abbreviations

WWW: World Wide Web
WAB: Web Accessibility Barriers
NTIA: National Telecommunications and Information Administration
W3C: WWW Consortium
WAI: Web Accessibility Initiative
WCAG: Web Content Accessibility Guidelines
WUI: Web Usability Index
NCI: National Cancer Institute
PDF: Portable Digital Format
ASP: Active Server Page
JSP: Java Server Page
XML: eXtensible Markup Language
ANOVA: ANalysis Of VAriance
NIH: National Institutes of Health
CHID: Combined Health Information Database
